# Alterations in expression of endometrial genes coding for proteins secreted into the uterine lumen during conceptus elongation in cattle

**DOI:** 10.1186/1471-2164-14-321

**Published:** 2013-05-10

**Authors:** Niamh Forde, Jai P Mehta, Paul A McGettigan, Solomon Mamo, Fuller W Bazer, Thomas E Spencer, Pat Lonergan

**Affiliations:** 1 School of Agriculture and Food Science, University College Dublin, Dublin, Ireland; 2 Conway Institute of Biomolecular and Biomedical Science, University College Dublin, Dublin, Ireland; 3 Department of Animal Science, Texas A&M University, College Station, Texas, 77843-2471, USA; 4 Center for Reproductive Biology, Department of Animal Sciences, Washington State University, Pullman, Washington, 99164-6353, USA; 5 Veterinary Sciences Centre, School of Agriculture and Food Science, University College Dublin, Belfield, Dublin 4, Ireland

**Keywords:** Uterus, Endometrium, Gene expression, Histotroph, Embryo, Progesterone, Estrous cycle, Pregnancy, Proteomics

## Abstract

**Background:**

We hypothesized that genes that are up-regulated in the uterine endometrium at the initiation of conceptus elongation in cattle, and that encode for secreted proteins, contribute to the composition of the uterine luminal fluid (ULF) and ultimately, drive conceptus elongation. The aims of this study were to: 1) screen endometrial transcriptomic data for genes that encode secreted proteins on Day 13; 2) determine temporal changes in the expression of these genes during the estrous cycle/early pregnancy; 3) determine if expression of these genes is affected by altered concentrations of progesterone (P4) *in vivo* and 4) determine if the protein products of these genes are detectable in ULF.

**Results:**

Of the fourteen candidate genes examined, quantitative real-time PCR analysis revealed the expression of *APOA1*, *ARSA*, *DCN*, *LCAT*, *MUC13*, *NCDN*, *NMN*, *NPNT*, *NXPH3*, *PENK*, *PLIN2* and *TINAGL1* was modulated in the endometrium (P<0.05) as the estrous cycle/early pregnancy progressed. *APOA1*, *DCN* and *NPNT* expression was higher in cyclic compared to pregnant heifers, and pregnancy increased (P<0.05) the expression of *LCAT*, *NCDN*, *NMN*, *PLIN2* and *TINAGL1*. The magnitude of the increase in expression of *APOA1*, *PENK* and *TINAGL1* on Day 13 was reduced (P<0.05) in heifers with low P4. Furthermore, low P4 decreased (P<0.05) the expression of *LCAT* and *NPNT* on Day 7, while an early increase (P<0.05) in the expression of *NXPH3* and *PLIN2* was observed in heifers with high P4. The protein products of 5 of the candidate genes (*APOA1, ARSA, LCAT, NCDN* and *PLIN*) were detected in the ULF on either Days 13, 16 or 19 of pregnancy.

**Conclusion:**

Using a candidate gene approach, we determined that both P4 concentration and the presence of the conceptus alter endometrial expression of *PLIN2*, *TINAGL1*, *NPNT*, *LCAT*, *NMN* and *APOA1*. Comparison of the expression profiles of these genes to proteins detected in ULF during conceptus elongation (i.e., Days 13 through 19) revealed the presence of APOA1, ARSA, LCAT, NCDN as well as members of the PLIN family of proteins that may play roles in driving conceptus elongation in cattle.

## Background

In cattle, the majority of pregnancy loss is attributed to early embryonic loss prior to maternal recognition of pregnancy [1,2], which occurs by approximately Day 16 following conception [3,4]. During this period, following successful fertilization, the developing embryo enters the uterus on Days 4 to 5 and forms a blastocyst by Day 7. Following hatching of the blastocyst from the zona pellucida (Day 9 to 10), the conceptus (embryo and associated extra-embryonic membranes) becomes ovoid in shape. Conceptus elongation is initiated by Days 13 to 14, and is characterized by a period of rapid growth of the trophectoderm cells that is completed by Days 19 to 20. Elongation of the conceptus requires molecules that are transported or secreted by the luminal (LE) and glandular (GE) epithelial cells of the uterine endometrium [5]. These secretions make up the uterine lumen fluid (ULF or histotroph), and include proteins, amino acids, lipids, glucose and ions, which are required for successful elongation, as evidenced by the failure of blastocysts to elongate *in vivo* in the uterine gland knock out ewe [6,7] and the inability to replicate elongation *in vitro* in cattle [8,9].

One of the pre-requisites for establishing a uterine environment capable of supporting conceptus elongation is an adequate post-ovulatory rise in circulating progesterone (P4) concentrations. In both cattle and sheep, elevated P4 in the post-ovulatory period results in advanced conceptus elongation [10-12]. As a consequence, the elongating conceptus produces more interferon-tau (IFNT) which is detectable in the uterine lumen [13,14] and which is associated with increased pregnancy rates [15-17]. The effects of P4 do not directly affect the developing embryo [18] but act indirectly by altering the transcriptome of the endometrium and subsequently the composition of the ULF [19-21].

One of the strategies employed to identify molecules that regulate conceptus elongation in ruminants has been to characterize the temporal changes in endometrial gene expression during the period when conceptus elongation occurs, and to infer from the localization of transcripts whether the proteins for which they encode contribute to the composition of the ULF. In sheep, genes localized to the uterine LE and superficial glandular epithelium (sGE) have been associated with 3 main classes of molecules: (i) proteins that are detectable in the uterine luminal fluid (ULF; *GlyCAM-1*[22], *CTSL*[23], *STC1*[24], *CST3*[25], *GRP*[26], and *IGFBP1*[27]), (ii) enzymes that produce constituents of the ULF (PTGS2 and HSD11B1, involved in prostaglandin [28] and cortisol [29] production, respectively) and (iii) active transporters of ULF molecules (*SLC2A1*, *SLC5A1*, *SLC1A5*, *SLC7A2* involved in amino acid [30,31], glucose [32] and ion transport). To date, detailed data on ULF composition in cattle are scarce, although some studies have detected the protein products of a small number of genes encoding for secreted proteins in the ULF including IGFBP1 [27], retinol binding protein [33,34], legumain and TIMP2 [35]. Caution in extrapolating the abundant data on ULF composition confirmed in sheep [36] to the bovine model is required. For example, despite the similarities in the transcriptomic changes that occur during the estrous cycle and early pregnancy, discrepancies in expression exist, as exemplified by *LGALS15*, which has a functional role in early pregnancy in sheep [37], but is not expressed in the bovine endometrium although is present in the bovine genome [38].

We have recently generated a considerable amount of data on the transcriptomic signature of the bovine endometrium before and during the initiation of conceptus elongation [39-42]. We hypothesize that genes that are up-regulated at the initiation of conceptus elongation, and that encode for secreted proteins, may contribute to the composition of the ULF and ultimately, drive conceptus elongation. With this in mind, the aims of this study were to: 1) screen endometrial transcriptomic data (generated by microarray and RNA sequencing) for genes that encode secreted proteins on Day 13 following estrus; 2) determine temporal changes in the expression of these genes during the estrous cycle/early pregnancy; 3) determine if expression of these genes is affected by altered concentrations of P4 *in vivo* that are known to impact conceptus elongation; and 4) determine if the protein products of these genes are detectable in ULF.

## Methods

All experimental procedures involving animals were licensed by the Department of Health and Children, Ireland, in accordance with the Cruelty to Animals Act (Ireland 1876) and the European Community Directive 86/609/EC and were sanctioned by the Animal Research Ethics Committee of University College Dublin. Unless otherwise stated, all chemicals and reagents were sourced from Sigma (Dublin, Ireland).

### Selection of candidate genes from transcriptomic data sets

To identify candidate genes from our gene expression data sets, two separate approaches were taken (Table 1). RNA sequencing (RNA-SEQ) data from cyclic heifers on Day 13 of the estrous cycle were screened to identify the most highly expressed transcripts in the endometrium that were not detectable in the conceptus at the corresponding stage of development [41,43] using a previously described approach [44]. Briefly, only transcripts detected in four out of five replicates for either the endometrium or conceptus, with an RPKM (reads per kilobase of exon per million mapped sequence reads) value of at least 0.5 were considered. The resulting list was screened to identify genes expressed only in cells of the endometrium (i.e., not in the conceptus) on Day 13. This list was then subjected to DAVID analysis (http://david.abcc.ncifcrf.gov/) to identify those genes that encoded for proteins assigned to the gene ontology terms ‘secreted’ or ‘extracellular’. Additional candidate genes were selected on the basis of a significant increase in expression on Day 13 compared to Day 7 of the estrous cycle [40,42].

**Table 1:** **Candidate genes selected for analysis on the basis of their increased expression on Day 13 as compared to Day 7 of the estrous cycle from reference**[42]**and RNA sequencing (RNA-SEQ) data of pregnant and cyclic endometria on Day 13 from reference**[41]**for the most abundant genes expressed in the endometria, but not detected in the conceptus**

**Gene name**	**Gene description**	**Gene ID/Affymetrix probe ID**	**Platform**	**Fold change (log2) on Day 13 v Day 7**	**Average TPM (Day 13)**	**Reference**
*DCN*	Decorin Precursor (Bone proteoglycan II)(PG-S2)	ENSBTAT00000004562	RNA-SEQ	N/A	1464.006	[44]
*MGP*	Bos taurus matrix Gla protein (MGP), mRNA.	ENSBTAT00000016414	RNA-SEQ	N/A	2371.502	[44]
*PENK*	Proenkephalin A Precursor [Contains Synenkephalin;Met-enkephalin(Opioid growth factor)(OGF);Met-enkephalin-Arg-Gly-Leu;Leu-enkephalin;Enkelytin;Met-enkephalin-Arg-Phe]	ENSBTAT00000006478	RNA-SEQ	N/A	907.056	[44]
*PLIN2*	Adipophilin (Adipose differentiation-related protein)(ADRP)	ENSBTAT00000047728	RNA-SEQ	N/A	62.58	[44]
*APOA1*	Apolipoprotein A-I	Bt.1229.1	Array	2.64	N/A	[45]
*ARSA*	Arylsulfatase A	Bt.1076.1	Array	3.14	N/A	[45]
*CYR61*	Cysteine-rich, angiogenic inducer, 61	Bt.22000.1	Array	2.87	N/A	[45]
*LCAT*	Lecithin-cholesterol acyltransferase	Bt.11126.3	Array	2.91	N/A	[45]
*MUC13*	Mucin 13, cell surface associated	Bt.12140.1	Array	26.72	N/A	[45]
*NMN*	Neuromedin N	Bt.10630.1	Array	2.72	N/A	[45]
*NCDN*	Neurochondrin	Bt.8806.1	Array	3.28	N/A	[45]
*NPNT*	Nephronectin	Bt.7393.1	Array	9.96	N/A	[45]
*NXPH3*	Neurexophilin 3	Bt.27342.1	Array	6.96	N/A	[45]
*TINAGL1*	Tubulointerstitial nephritis antigen-like 1	Bt.1300.1	Array	12.84	N/A	[45]

All genes selected for analysis encoded for secreted or extracellular proteins as determined by DAVID analysis (http://david.abcc.ncifcrf.gov/).

### Experiment 1: temporal changes in endometrial expression of candidate genes from cyclic and pregnant heifers during the pre-implantation period of early pregnancy

The estrous cycles of cross-bred beef heifers (n = 100, predominantly Charolais and Limousin cross) were synchronized by insertion of an intravaginal controlled internal drug release (CIDR) device (1.94 g P4; InterAg, Hamilton, New Zealand) for 8 days. One day prior to CIDR removal each heifer received an intramuscular injection of a prostaglandin F_2α_ (PG) analogue (Estrumate, Shering-Plough Animal Health, Hertfordshire, UK; 2 ml equivalent to 0.5 mg cloprostenol). Only those heifers observed in standing estrus (Day 0) were utilized further and were assigned randomly to either an inseminated group (Pregnant, P: n=59) or an un-inseminated cyclic control group (C: n=24). Heifers were slaughtered on either Day 7, 10, 13 or 16 (C group) of the estrous cycle or Day 7, 10, 13, 16 or 19 (P group) following estrus and insemination. These stages correspond in pregnant heifers to blastocyst formation, blastocyst hatching, initiation of conceptus elongation, maternal recognition of pregnancy and initiation of implantation, respectively. Thirty minutes after slaughter the uterine horn ipsilateral to the corpus luteum (CL) was flushed with 20 ml of 10 mM Tris (pH 7.2). ULF was centrifuged at 3,000 g for 10 min, supernatant removed and snap frozen in liquid nitrogen prior to proteomic analysis. In the inseminated group, only those reproductive tracts from which an embryo/conceptus at the expected stage of development was recovered (Day 7: blastocyst; Day 10: hatched blastocyst; Day 13: ovoid ; Days 16 and 19: filamentous conceptus) were processed further. Intercaruncular endometrial tissue was dissected free from the underlying myometrium and snap-frozen in liquid nitrogen for RNA extraction and quantitative real-time PCR (qPCR) analysis.

### Experiment 2: P4 effects on endometrial gene expression of candidate genes

In order to investigate the effect of circulating P4 on the expression of these candidate genes, we examined the endometrium of heifers with low, normal and high P4 from previously generated tissue samples. The estrous cycles of cross-bred beef heifers were synchronized as described in Experiment 1 and those observed in standing estrus (n=52) were randomly assigned to one of three treatments: (i) cyclic heifers normal P4, no treatment (n=5); (ii) cyclic heifers, high P4 (n=5), receiving an intravaginal P4 device on Day 3 of the estrous cycle to increase concentrations of P4 in blood [11]; and (iii) cyclic heifers, low P4 (n=5) receiving 3 intramuscular injections of a PG analogue (Estrumate) on Days 3, 3.5 and 4 of the estrous cycle to reduce P4 output from the CL [40]. The progesterone profiles of animals on these treatments have been extensively reported by us in previous papers [11,18,39,40,42,45]. Following slaughter, intercaruncular endometrial tissue from the tip of the uterine horn ipsilateral to the CL was recovered, snap frozen in liquid nitrogen for subsequent RNA extraction and analyzed using qPCR for selected genes.

### Quantitative real-time PCR analysis (qPCR)

All qPCR analyses were performed as previously described [42]. Total RNA was extracted from 100 mg of intercaruncular endometrial tissue using Trizol reagent as per manufacturer’s instructions. Both RNA clean-up and on-column DNase treatment were performed (Qiagen, Crawley, Sussex, UK) and the subsequent RNA was analyzed for both quality and quantity using the Agilent Bioanalyzer (Agilent Technologies, Santa Clara, CA, USA) and Nanodrop 1000 (Thermo Fischer Scientific, DE, USA), respectively. One microgram of total RNA was converted to complementary DNA (cDNA) using Superscript III (Applied Biosystems, Foster City, CA, USA) and random hexamers as per manufacturer’s instructions. All primers were designed using Primer-BLAST software (http://www.ncbi.nlm.nih.gov/tools/primer-blast/) to span exon-exon boundaries where possible. Each qPCR reaction was carried out in duplicate with 50 ng of cDNA, optimized primer concentrations (Table 2) and 7.5 μl FAST Sybrgreen mastermix (Applied Biosystems, Foster City, CA, USA) in a final reaction volume of 15 μl. All reactions were carried out using the 7500 Fast Real-Time PCR System (Applied Biosystems, Foster City, CA, USA) with the following cycling conditions: 2 min at 50°C; 10 min at 95°C; and 40 cycles of 95°C for 15 sec and 60°C for 1 min. A dissociation curve was included in each run to ensure specificity of amplification, while a standard curve was included for each gene of interest as well as for the normalizer genes (*ACTB* and *RPL19*) to determine primer efficiencies. All raw cycle threshold values were then imported into qbase^plus^ software (Biogazelle, Zwijnaarde, Belgium) used to calibrate and normalize data, as well as calculate expression values for each gene in arbitrary units (CNRQ).

**Table 2:** Primer information used for quantitative real time PCR analysis of candidate genes

**Entrez gene symbol**	**gene name**	**Accession number**	**Forward primer sequence**	**Reverse primer sequence**	**Product length (bp)**
*ACTB*	Actin, beta	NM_173979.3	CGCCATGGATGATGATATTGC	AAGCCGGCCTTGCACAT	66
*APOA1*	Apolipoprotein A-I	NM_174242.3	GCTGGCCATTGAGGTCACCCAC	GCTGCCAGAAATGCCGAGCCT	103
*ARSA*	Arylsulfatase A	NM_001075205.1	GCCTTTGCCCGCGACCTCAT	TGGGTGTGGTGGGAGGCGTA	80
*CYR61*	Cysteine-rich, angiogenic inducer, 61	NM_001034340.2	TGCAGAGCTCAGTCGGAGGGC	GGCGCCGTCGATACATGTGC	111
*DCN*	Decorin	NM_173906.4	ACTCTTCAGGAGCTGCGTGTCCA	GCGGGTTGGTGCCAAGTTCTACG	106
*LCAT*	Lecithin-cholesterol acyltransferase	NM_001046069.2	CGGCCCGTCATCCTCGTGCC	AAGTCCTCCGTCTTGCGGTAGCA	104
*MGP*	Matrix Gla protein	NM_174707.2	GCAAAAGCCCAAGAGAGAATCCGA	ACACCATGGCATAGCGTTCGCA	103
*MUC13*	Mucin 13, cell surface associated	XM_002702427.2	ACGGGCTGGTGAGACCAAAACC	GCAGTCAGCTGTCCCGTTGC	116
*NCDN*	Neurochondrin	NM_001045931.1	TCTCCAGCTCTGCAGGGGACG	GGCCACGTGGGATTGCGACA	81
*NMN*	Neuromedin N	NM_173945.4	GAGCCCCCTTCAGCCTGTTCC	GCCAGAAGAATCATGCACACCAGC	104
*NPNT*	Nephronectin	XM_591031.5	TCTCAGCAGCCAAAGGCCCG	CTGACAGGCACAGGTCCCCT	90
*NXPH3*	Neurexophilin 3	NM_001192824.1	ATGACGGTCCGCCAGGCTCA	CCGCTTTCGAGGCATCCGGG	80
*PENK*	Proenkephalin	NM_174141.2	GACGCCGAGGACCGCGAGAG	CCATGGGGTTGCCGCTGTTCGG	120
*PLIN2*	Periliphin 2	NM_173980.2	TCTTCGCGCTTGGGCGTCTG	CCACCCTGGTCACCACACTCAGTT	101
*RPL19*	Ribosomal protein L19	NM_001040516.1	GAAAGGCAGGCATATGGGTA	TCATCCTCCTCATCCAGGTT	86
*TINAGL1*	Tubulointerstitial nephritis antigen-like 1	XM_003585097.1	CTCGGGAGGCCGGAGCGATA	GTCAGGCAGCGTCTCCTCGC	81

All primers were used at a concentration of 300 nM in a final reaction volume of 15 μl. A dissociation curve was included to ensure specificity of each primer pair.

### Proteomic analysis of uterine luminal fluid during the peri-implantation period of pregnancy

Nano LC MS/MS analysis was carried out on ULF from pregnant heifers on Days 13 and 16 to identify proteins involved in conceptus elongation (Applied Biomics, Inc, Hayward, CA, USA). Samples (pool of n=5 heifers on Day 13 and Day 16) were exchanged into 50 mM ammonium bicarbonate buffer, DTT added to a final concentration of 10 mM, and samples were incubated at 60°C for 30 min followed by cooling to room temperature (RT). Iodoacetamide was added to a final concentration of 10 mM and incubated in the dark for 30 min at RT. A tryptic digestion was performed at 37°C overnight. Nano LC was carried out using a Dionex Ultimate 3000 (Milford, MA, USA). Tryptic peptides were loaded into an α-Precolumn Cartridge and separated using an acetonitrile gradient (ranging from 5% to 60%) on the Nano LC column. Fractions were collected at 20 sec intervals followed by Mass Spectrometry analysis on AB SCIEX TOF/TOF™ 5800 System (AB SCIEX, Framingham, MA, USA). Mass spectra were acquired in reflectron positive ion mode. TOF/TOF tandem MS fragmentation spectra were acquired for each ion, averaging 4000 laser shots per fragmentation spectrum (excluding trypsin autolytic peptides and other known background ions). Identification of the resulting peptide mass and the associated fragmentation spectra were submitted to GPS Explorer workstation equipped with MASCOT search engine (Matrix Science, London, UK) to search the non-redundant database of National Center for Biotechnology Information (NCBInr). Searches were performed without constraining protein molecular weight or isoelectric point, with variable carbamidomethylation of cysteine and oxidation of methionine residues, and with one missed cleavage also being allowed in the search parameters.

iTRAQ analysis was carried out on ULF recovered from pregnant heifers on Days 13, 16 and 19 (n=4 per day) to identify quantitative changes in the protein content (Proteome factory, Berlin, Germany). Two ml of each individual sample was precipitated in 10 ml of 100% EtOH overnight, the resulting pellet was washed twice and resuspended in 40 μl of lysis buffer and centrifuged at 13,000 g. The supernatant was incubated for 30 min at RT with 10 mM iodoacetamide in the dark and the resulting protein concentration was determined by Bradford assay. One hundred micorgrammes of total protein was subjected to trypsin digestion (Promega, Mannhein, Germany) at 37°C overnight with additional trypsin added and the reaction continued for a further 3 h. The resulting peptides were acidified with formic acid (pH 2.0), desalted with Macro spin tips containing Vydac C18 material (Nest group, Southborough, MA, USA) and lyophilized. The lyophilized samples were dissolved in 45 μl of iTraq buffer (AB SCIEX, Framingham, MA, USA) and 30 μl of each sample were reacted with appropriate iTraq reagent for 2 h at RT as per the manufacturer’s protocol. The reaction was stopped with 50 μl of 20% formic acid, pH 2.0 and dried by lyophilisation. Strong cation exchange (SCX) was performed on a PolySULFOETHYL A column (200 mm × 2.1 mm, 5 μm, 200 Å, PolyLC, Columbia, MD, USA) using an Agilent 1100 HPLC system (Agilent, Karlsruhe, Germany) with 18 fractions collected per sample. Protein identification and quantification of iTRAQ reporter ions was performed using nanoLC‒ESIMS/MS which consisted of an Agilent 1100 nanoLC system (Agilent, Karlsruhe, Germany), PicoTip emitter (New Objective, Langhorne, PA, USA) and a QExactive quadrupole‒Orbitrap mass spectrometer (ThermoFisher Scientific, Bremen, Germany). The dried SCX peptide fractions were resuspended in 80 μl of MilliQ water containing 0.1% formic acid and 1% acetonitrile. After trapping 40 μl of each sample the peptides were desalted for 5 min on an enrichment column (Zorbax SB C18, 0.3 mm × 5 mm, Agilent, Karlsruhe, Germany) using a solution of 1% acetonitrile and 0.1% formic acid solution. All peptides were separated on a Zorbax 300 SB C18, 75 μm × 150 mm column (Agilent, Karlsruhe, Germany) for 110 min, using an acetonitrile gradient containing 5-25% acetonitrile in 0.1% formic acid. The mass spectrometer was operated in a data‒dependent mode by subjecting the ten most abundant ions of each survey spectrum (nominal resolution 35.000) to HCD fragmentation (normalized collision energy at 40%, Resolution 17.500). MSMS peak lists were extracted to mascot generic format files and searched by the Mascot search algorithm against the bovine IPI database that has been curated from duplicate entries. The mass tolerance was set to 5 ppm for peptide masses and 0.02 Da for fragment ions. Quantitative information was obtained with the Protein ratio type set to “weighted” and normalization to “summed intensities”.

### Data analysis

All data were analysed using the statistical package SAS (SAS Institute Inc., Cary, NC). For gene expression analysis, the log of the CNRQ values in arbitrary units was used and analysis was performed using the general linear model procedure (PROC GLM) with day, pregnancy status and/or P4 status, when appropriate, as the main effects. Treatment effects on gene expression were separated by Tukey’s test and a p value of ≤ 0.05 was considered significant.

## Results

### Identification of candidate genes from large-scale transcriptomic data sets

From the RNA-SEQ data, a total of 208 distinct transcripts with an extracellular localization were uniquely detected in the endometrium (Additional file 1: Table S1). Three genes had RPKM values of greater than 1,000 in the endometrium (matrix gamma-carboxyglutamic acid, *MGP* 1732±144 RPKM; proenkephalin, *PENK* 1278±336 RPKM; and decorin, *DCN* 1048±136 RPKM) and were chosen for further analysis. Fourteen genes had expression values in the endometrium of between 100 and 1,000 RPKM (*AGT*, *APOD*, *AZGP1*, *CLEC3B*, *COL1A2*, *COL3A1*, *IP1*, *ISG15*, *LUM*, *PDZK1*, *PIP*, *PLAT*, *PRSS16*, *SAA1* and *SPARCL1*). Of the remaining genes, 71 had expression levels between 10 and 100 RPKM, while 120 genes had expression of < 10 RPKM in the endometrium of cyclic heifers on Day 13 (Additional file 1: Table S1). None of these genes were expressed in the conceptus at levels detectable by RNA-SEQ (<0.5 RPKM). Screening of microarray data indicated increased expression on Day 13 of an additional 10 candidate genes that encode for secreted or extracellular proteins including apolipoprotein A-1 (*APOA1*), arylsulfatase A (*ARSA*), cysteine-rich, angiogenic inducer, 61 (*CYR61*), lecithin-cholesterol acyltransferase (*LCAT*), mucin 13, cell surface associated (*MUC13*), neuromedin N (*NMN*), neurochondrin (*NCDN*), nephronectin (*NPNT*), neurexophilin 3 (*NXPH3*) and tubulointerstitial nephritis antigen like-1 (*TINAGL1*). These were selected as they have not been previously interrogated in the context of conceptus elongation in cattle.

### Changes in endometrial gene expression throughout the estrous cycle and early pregnancy

**Figure 1:**
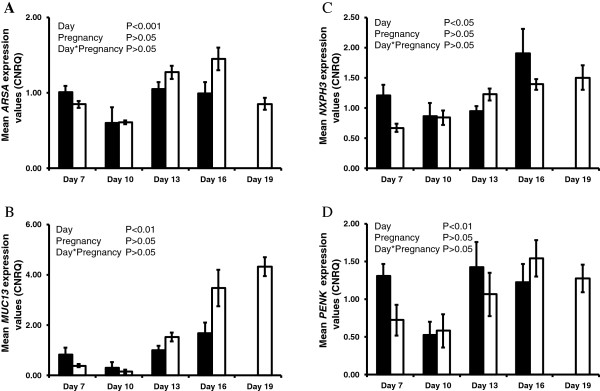
**qPCR analysis of candidate genes in the endometrium significantly affected by day of the estrous cycle and early pregnancy. A**-**D** Average calibrated, normalized relative expression values in arbitrary units (CNRQ ± SEM) determined by qPCR analysis of the endometrium from cyclic (black bars) and pregnant (open bars) heifers (n=5 per treatment per time-point) throughout the estrous cycle and early pregnancy.

**Figure 2:**
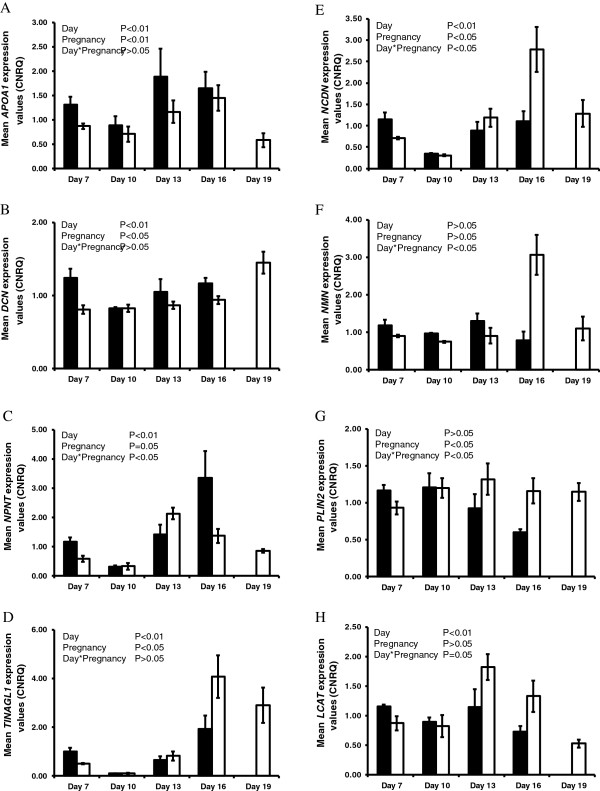
**qPCR analysis of candidate genes in the endometrium significantly affected by pregnancy status. A**-**H** Average calibrated, normalized relative expression values in arbitrary units (CNRQ ± SEM) as determined by qPCR analysis of the endometria from cyclic (black bars) and pregnant (open bars) heifers (n=5 per treatment per time-point) throughout the estrous cycle and early pregnancy.

The endometrial expression of *ARSA*, *MUC13*, *NXPH3* and *PENK* increased (P<0.05, day effect) as the estrous cycle/early pregnancy progressed (Figure 1); however, pregnancy status had no significant effect on their expression. No effects of day, pregnancy or their interactions were detected for *CYR61* or *MGP* (Table 3). The expression of *APOA1*, *DCN*, *LCAT*, *NCDN*, *NMN*, *NPNT*, *PLIN2* and *TINAGL1* was modulated (P<0.05, day effect) as the estrous cycle/early pregnancy progressed (Figure 2). In addition, *APOA1*, *DCN* and *NPNT* expression was higher in the endometrium of cyclic compared to pregnant heifers, while pregnancy increased (P<0.05) the expression of *LCAT*, *NCDN*, *NMN*, *PLIN2* and *TINAGL1* in the endometrium.

**Table 3:** A summary of the overall effects of day, pregnancy status and progesterone (P4) concentrations on endometrial expression of selected genes that encode for secreted proteins

**Gene name**	**Gene description**	**Day**	**Pregnancy**	Day *Pregnancy	**P4**	**Protein detected in ULF**
*APOA1*	Apolipoprotein A-I	*	*	-	*	YES
*ARSA*	Arylsulfatase A	***	-	-	-	YES
*CYR61*	Cysteine-rich, angiogenic inducer, 61	-	-	-	-	ND
*DCN*	Decorin Precursor (Bone proteoglycan II)(PG-S2)	**	*	-	-	ND
*LCAT*	Lecithin-cholesterol acyltransferase	**	-	*	*	YES
*MGP*	Bos taurus matrix Gla protein (MGP), mRNA.	-	-	-	*	ND
*MUC13*	Mucin 13, cell surface associated	**	-	-	-	ND
*NCDN*	Neurochondrin	**	*	*	-	YES
*NMN*	Neuromedin N	-	-	*	*	ND
*NPNT*	Nephronectin	**	*	*	*	ND
*NXPH3*	Neurexophilin 3	*	-	-	*	ND
*PENK*	Proenkephalin A Precursor [Contains Synenkephalin;Met-enkephalin(Opioid growth factor)(OGF);Met-enkephalin-Arg-Gly-Leu;Leu-enkephalin;Enkelytin;Met-enkephalin-Arg-Phe]	**	-	-	*	ND
*PLIN2*	Adipophilin (Adipose differentiation-related protein)(ADRP)	-	*	*	*	YES
*TINAGL1*	Tubulointerstitial nephritis antigen-like 1	**	*	-	*	ND

Protein encoded for by these genes were detected in uterine luminal fluid during the period of conceptus elongation (Day 13) through to initiation of implantation (Day 19) by either nano LC MS/MS or iTraq analyses. Significance is set at P<0.05 (*), P<0.01 (**) or P<0.001 (***). – denotes no significant effect of a given treatment. ND = protein product not detected by either method.

### Regulation of candidate gene expression in the endometrium by altered progesterone concentrations in vivo

**Figure 3:**
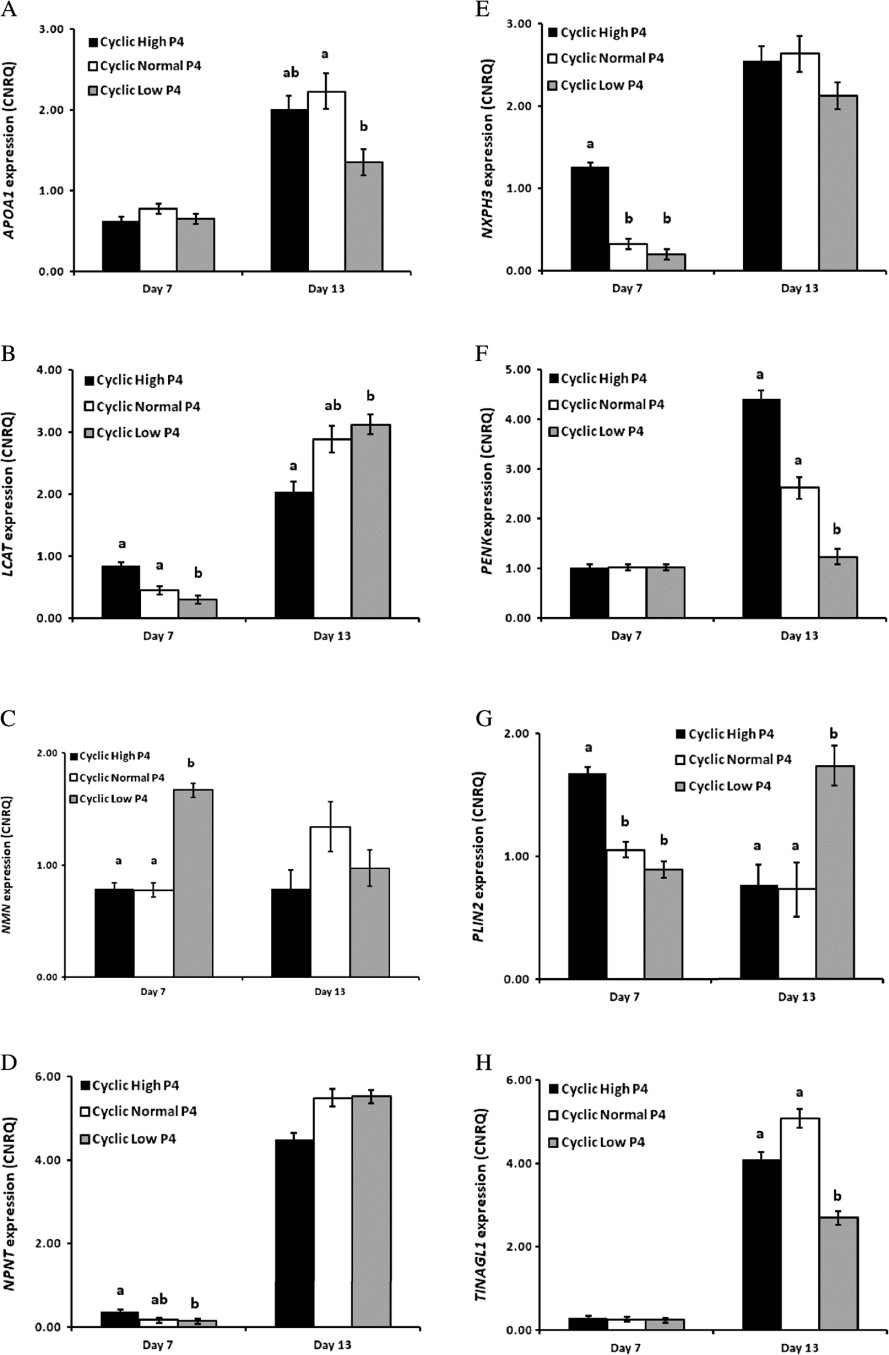
**qPCR analysis of candidate genes in the endometrium significantly affected by progesterone. A**-**H** Average calibrated, normalized, relative expression values (CNRQ ± SEM) as determined by qPCR in the endometrium of heifers with high (black bars), normal (white bars) and low (gray bars) circulating concentrations of P4 on either Day 7 or Day 13 of the estrous cycle. Significant differences (P<0.05) in gene expression values between treatments on a given day are identified with a, b, or c.

High and low concentrations of circulating P4 were associated with four main patterns of gene expression in the endometrium. The expression of *APOA1*, *PENK* and *TINAGL1* was similar among treatment groups on Day 7; however, the magnitude of the normal increase in expression of these genes on Day 13 was reduced (P<0.05) in heifers with low P4 (Figure 3A, F & H). In contrast, low P4 decreased (P<0.05) the expression of *LCAT* and *NPNT* on Day 7 only (Figure 3B & D), while an early increase (P<0.05) in the expression of *NXPH3* and *PLIN2* was observed in heifers with high P4 (Figure 3E & G). *NMN* expression was higher (P<0.05) in low P4 heifers on Day 7 compared to high and normal P4 groups (Figure 3C), while the expression of *ARSA*, *DCN*, *MUC13* and *NCDN* was not affected by altered concentrations of circulating P4 (data not shown).

### Protein abundance in the ULF during the peri-implantation period of early pregnancy

**Figure 4:**
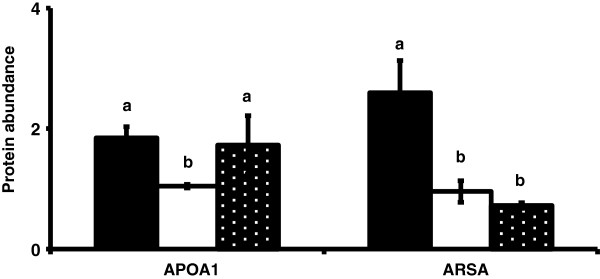
**Protein abundance in the uterine luminal fluid for APOA1 and ARSA on Days 13 (solid bars), 16 (open bars) and 19 (black bars, white stipple) of pregnancy based on results from iTraq analysis.** Differences (P<0.05) in protein abundance between days are represented with a, b, or c.

To further test the hypothesis that these molecules play a role in conceptus elongation, the presence of the protein products of these genes in ULF during the peri-implantation period of pregnancy was examined. Of the 12 candidate genes which encode for putatively secreted proteins and whose expression was modulated in the endometrium during the estrous cycle and early pregnancy, 5 were detected by either iTraq or nano LC MS/MS analysis of ULF collected on either Days 13, 16 or 19 of pregnancy. Using iTraq, LCAT was detected in ULF of pregnant heifers on Day 13 of pregnancy, while NCDN and PLIN3 were detected on Days 16 and 19. Quantitative changes in their abundance were not detected, however. PLIN1 was detected on Days 13 and 16 of pregnancy by nano LC MS/MS. In contrast APOA1 and ARSA proteins were detected in ULF using both iTraq and nano LC MS/MS on all days of pregnancy examined. The abundance of APOA1 and ARSA declined (P<0.05) from Day 13 to Day 16 (Figure 4).

## Discussion

In cattle, as in other ruminants, the transition from a blastocyst to a fully elongated conceptus is one of the major morphological events that take place during the peri-implantation period of pregnancy. This process is a maternally-driven event, as elongation does not occur *in vivo* in the absence of uterine gland secretions in sheep [6], and has yet to be recapitulated *in vitro* in cattle [8,9]. Transfer of *in vitro* derived blastocysts [18] or trophoblastic vesicles [46] into the uterus of synchronized recipients results in successful conceptus elongation; ULF must therefore contain molecules that drive conceptus elongation. One approach to predicting which proteins are present in ULF is to examine the endometrial expression of selected genes that encode for proteins that are known to be secreted or extracellular in nature. If expression of these genes is modulated during early pregnancy and/or by altered concentrations of P4 *in vivo* (which have demonstrable effects on the elongation of the conceptus), it is plausible that they are candidate genes likely to influence conceptus elongation. Using this candidate gene approach, we found that expression of *ARSA*, *MUC13*, *NXPH3* and *PENK* increased with advancing stages of the estrous cycle and early pregnancy. Similarly, *LCAT*, *NCDN*, *NMN*, *PLIN2* and *TINAGL1* expression increased during the later luteal phase, with the presence of the conceptus significantly increasing their expression. In contrast, *APOA1*, *DCN* and *NPNT* expression decreased in pregnant compared with cyclic heifers. A delay in the postovulatory increase in circulating concentrations of P4 delayed the increase in expression of *LCAT* and *NPNT* on Day 7 and *APOA1*, *PENK* and *TINAGL1* on Day 13, the day on which conceptus elongation is initiated. Finally, comparison of this list of candidate genes with the protein content of ULF of pregnant heifers during conceptus elongation (Days 13 through 19) indicated the presence of APOA1, ARSA, LCAT, NCDN as well as members of the PLIN family, lending validity to this candidate gene expression approach.

In cattle, two of the main molecules that affect endometrial gene expression are circulating concentrations of P4 and conceptus-derived IFNT, both of which can affect the composition of the ULF and alter the rate of conceptus elongation. The comparison of gene expression in the endometrium and proteins present in ULF identified four proteins (APOA1, ARSA, LCAT and NCDN) from 14 candidate genes, while two members of the perilipin family were detected. At first glance, the number of proteins detected in ULF in comparison to the number of candidate genes may seem low. However, it is important to note that there is at least an order of magnitude in the difference between the number of total transcripts detectable in the endometrium by RNA-SEQ (~21,000 transcripts: [41]) versus the number of total proteins detected in ULF by proteomics [47]. In addition, analysis of gene expression was performed on endometrial homogenates which contain a heterogenous cell population. It is possible that, despite their status as genes that encode for extracellular/secreted proteins, their expression may be predominantly stromal in nature, similar to the expression of *HGF*[48] and *FGF10*[49] in the sheep endometrium. As such, the protein products of these genes may not be secreted into ULF, but may act in a more autocrine or paracrine manner within the endometrium.

The patterns of expression of *ARSA*, *MUC13*, *NXPH3* and *PENK* were similar in endometria from both pregnant and cyclic heifers. *MUC13* exhibited minimal expression on Day 7 and 10, while *ARSA*, *NXPH3* and *PENK* expression decreased on Day 10, but increased significantly during the late luteal phase of the estrous cycle and as pregnancy progressed. Given that the expression of these genes increased during the luteal phase of the estrous cycle and early pregnancy, we propose that they play a role in establishing uterine receptivity to implantation. Although the protein products of *NXPH3* or *PENK* were not detected in ULF, the increased mRNA abundance for these genes in endometria of P4-supplemented heifers strongly suggests that they contribute to advancing conceptus elongation after both artificial insemination [11] and embryo transfer [18].

In contrast, endometrial expression of *APOA1*, *DCN* and *NPNT* decreased in to the presence of the developing conceptus. Expression of both *APOA1* and *NPNT* was modulated by P4, with a delay on Day 13 in *APOA1* expression, while P4 supplementation resulted in an early increase in *NPNT* on Day 7. We propose that the decreased expression of these genes in endometria in the peri-implantation period of pregnancy, may be a pre-requisite for establishing uterine receptivity to implantation, similar to that observed for nuclear P4 receptors in all species studied [50] as well as MUC1 in sheep [51].

The modulation of circulating concentrations of P4 in cattle and sheep has demonstrable effects on gene expression in the endometrium and timing of conceptus elongation. In cattle, this results in altered trajectories of conceptus elongation on Day 14 following embryo transfer on Day 7. Exogenous supplementation of P4 advances conceptus elongation [18]; conversely, when P4 output from the CL is diminished [40], conceptus elongation is delayed. High circulating concentrations of P4 advanced the normal increase of *NXPH3* expression on Day 7 of the estrous cycle, while in heifers with low P4 the normal expression level of *NPNT* was decreased. In addition, decreased expression of *APOA1* and *PENK* on Day 13 in heifers with low P4 and delayed conceptus elongation suggests that these endometrial genes and their protein products contribute to the process of conceptus elongation. This hypothesis is supported by evidence for the presence of APOA1 protein in ULF on both Day 13 and 16.

Of the 14 candidate genes examined, changes in expression of *NMN*, *PLIN2* and *TINAGL1* were of particular interest. Neurotensin (NT) was first isolated from the bovine hypothalamus [52] and small intestine [53] and NMN protein, along with NT is derived from the same precursor peptide with the resulting active form of the peptides (i.e., NT or NMN) being dependent of the site of cleavage of the pro-peptide (reviewed by [54]). Interestingly, the predominant forms of the processed peptides are tissue-specific, e.g., the long form of NMN is the predominant form in the gut [55]. *NMN* expression was similar in pregnant and cyclic heifers up to Day 16 when pregnancy recognition occurs and was modulated by low circulating concentrations of P4. NMN protein was not detected in ULF, but a protein that degrades NMN, aminopeptidase M [56], has been detected in ULF [47]. This may explain in part why NMN protein was not detected and, given its expression pattern, may suggest that NMN is involved in the pregnancy recognition response in the endometrium rather than conceptus elongation itself.

TINAGL1 is a matri-cellular protein that can interact with both extracellular matrix proteins and cell surface receptors. In mice, TINAGL1 is expressed by the trophectoderm and interacts with laminin 1 [57]. TINAGL1 expression increases in the endometrium of pregnant mice as well as at the sites of implantation where the abundance of protein increases during the later stages of implantation [58]. In addition, TINAGL1 has been linked to the integrins ITGA5 and ITGB1 in the decidua [58]. In the current study, *TINAGL1* expression was minimal from Day 7 to 13, but increased on Day 16 and to a greater extent in pregnant than cyclic heifers. This is interesting as there is basal expression when the uterus is not receptive to implantation (i.e., when PGR is expressed by uterine LE and GE). *TINAGL1* expression increases after uterine receptivity to implantation is established, but interestingly, its expression on Day 13, when conceptus elongation is initiated, is minimal. Moreover, TINAGL1 protein is not detectable in ULF. Thus, *TINALG1* in the endometrium of cattle is not directly involved in conceptus elongation, but instead plays a role in implantation as evidenced by its increase in expression in pregnant heifers on Days 16 and 19, similar to its role in mice.

PLIN2 (a.k.a. ADRP) protein is involved in lipid droplet formation and, in particular, the intracellular storage of triaclyglycerols (TAG: reviewed by [59]). It is also detectable in milk lipid droplets secreted from mammary epithelial cells in a variety of species including humans, rats and cattle [60]. Triaclyglycerols have been detected in the ULF of cattle [61] and *in vitro* TAG is utilized as an energy source by the embryo up to the blastocyst stage of development [62]. In both breast cancer and leiomyoma human cell lines, the *PLIN2* gene has a PGR response element in its promoter region. Given its modulation by P4 *in vivo* and its increased expression in pregnant heifers likely due to IFNT [63], as well as detection of PLIN2 family members in ULF of cattle, it is likely that PLIN2 contributes to the secretion and /or transport of TAG into the ULF as an energy source for the developing conceptus during elongation.

## Conclusion

Using a candidate gene approach, we determined that the expression of *PENK* is affected by altered concentrations of P4 in blood while pregnancy increases and decreases the expression of *NCDN* and *DCN,* respectively. In addition, we determined that both P4 manipulation and the presence of the conceptus alter endometrial expression of *PLIN2*, *TINAGL1*, *NPNT*, *LCAT*, *NMN* and *APOA1*. Comparison of the expression profiles of these genes to proteins detected in ULF during conceptus elongation (i.e., Days 13 through 19) revealed the presence of APOA1, ARSA, LCAT, NCDN as well as members of the PLIN family of proteins that may play roles in driving conceptus elongation in cattle.

## Competing interests

The authors declare that they have no competing interests.

## Authors’ contributions

NF and PL performed the sample collection. NF performed the laboratory analysis of the endometrium/uterine samples. SM performed gene expression analysis on the embryo. JPM, PMcG and NF performed data analysis. NF, FWB, TES and PL were involved in the conception and design of the study. NF drafted the manuscript. FWB, TES and PL critically revised the manuscript. All authors read, commented and approved the final version of the manuscript.

## Supplementary Material

Additional file 1: Table S1 Average expression values in transcripts per million (TPM±SEM) for genes with a cellular component of extracellular space in the endometrium of cyclic heifers on Day 13 of the estrous cycle. No detecteble expression values for these genes was observed in the ovoid conceputs at the same stage following estrus (Day 13). Click here for file
